# Causal Relationship of Coronary Artery Calcium on Myocardial Infarction and Preventive Effect of Antiplatelet Therapy

**DOI:** 10.3389/fcvm.2022.871267

**Published:** 2022-04-27

**Authors:** Thosaphol Limpijankit, Sutipong Jongjirasiri, Nattawut Unwanatham, Sasivimol Rattanasiri, Ammarin Thakkinstian, Jiraporn Laothamatas

**Affiliations:** ^1^Division of Cardiology, Department of Medicine, Faculty of Medicine Ramathibodi Hospital, Mahidol University, Ratchathewi, Thailand; ^2^Department of Radiology, Faculty of Medicine, Ramathibodi Hospital, Mahidol University, Ratchathewi, Thailand; ^3^Department of Clinical Epidemiology and Biostatistics, Faculty of Medicine Ramathibodi Hospital, Mahidol University, Ratchathewi, Thailand; ^4^Faculty of Heath Science Technology, Chulabhorn Royal Academy, Bangkok, Thailand

**Keywords:** myocardial infarction, coronary artery calcium, antiplatelet therapy, aspirin, coronary computed tomographic angiography

## Abstract

**Background:**

The role of coronary artery calcium score (CACS) to guide antiplatelet therapy in order to prevent myocardial infarction (MI) is still uncertain. This study aimed to find the causal relationship of CACS on MI and preventive effect of antiplatelet therapy.

**Methods:**

From 2005 to 2013, all patients with cardiovascular risk factors or symptoms of suspected CAD underwent coronary computed tomography. CACSs were measured using Agatston method and stratified into 4 groups: 0, 1–99, 100–399, and ≥ 400. Antiplatelet therapy was prescribed following physician discretion. Outcomes of interest were MI and bleeding. A mediation analysis was applied to find association pathways. CACS was considered as an independent variable, whereas antiplatelet therapy was considered as a mediator and MI considered the outcome of interest.

**Results:**

A total of 7,849 subjects were enrolled. During an average of 9.9 ± 2.4 years follow-up, MI and bleeding events occurred in 2.24% (*n* = 176) and 2.82% (*n* = 221) of subjects, respectively. CACSs 100–399 and CAC ≥ 400 were significantly associated with the development of MI [OR 3.14 (1.72, 5.72), and OR 3.22 (1.66, 6.25), respectively, *p* < 0.001]. Antiplatelet therapy reduced the risk of MI of these corresponding CAC groups with ORs of 0.60 (0.41, 0.78) and 0.56 (0.34, 0.77), *p* < 0.001]. A risk of bleeding was associated with antiplatelet therapy (only aspirin), anticoagulant, hypertension, male gender and old age.

**Conclusion:**

CACS was associated with the development of future MI. The preventive effect of antiplatelet therapy was clearly demonstrated in subjects with CACSs equal to or above 100, but this benefit was partially offset by an increased risk of bleeding.

## Introduction

The most serious and devastating clinical manifestation of coronary artery disease (CAD) is myocardial infarction (MI) (1). At least 25% of patients who developed MI and sudden death had no previous warning symptoms (2). Therefore, identification of asymptomatic individuals who are at greater risk of MI is important for the implementation of primary preventive strategies.

Coronary artery calcium score (CACS), a non-invasive quantitative assessment using computed tomography (CT), is associated with the presence and extent of subclinical atherosclerotic plaque (3) and has proven to be a powerful predictor of future MI (4). Previous studies also have shown a strong association between CACS and major cardiovascular (CV) outcomes in asymptomatic people (5–7). The CACS adds to risk assessment beyond the traditional atherosclerotic cardiovascular disease (ASCVD) risk factors (8, 9). Current clinical practice guidelines consider CACS to be a potential prognostic tool for prediction of CV events and useful in personalizing allocation of statin therapy (10, 11). There are preliminary findings which suggest that CACS may also help guide decisions for initiating (or deferring) antiplatelet therapy for primary prevention (12, 13).

Antiplatelet therapy, mainly aspirin, has been demonstrated to prevent the first episode of MI (14). The role of aspirin in the primary prevention of ASCVD is currently controversial. Three landmark randomized controlled trials (15–17) and two large meta-analyses (18, 19) have suggested no or marginal benefit of low-dose aspirin for the primary prevention of ASCVD events, while carrying a significant bleeding risk. Based on this evidence, aspirin was downgraded from a class I to a weak class IIb recommendation for primary prevention in the 2019 American College of Cardiology/American Heart Association (ACC/AHA) guideline (20). This recent recommendation is that aspirin may be considered in selected adults, 40–70 years of age, who are at higher CV risk, but not at increased bleeding risk. However, it remains unclear how to identify high risk patients who are likely to derive a net benefit from aspirin therapy when used for primary prevention.

Similar to the CACS guidance of statin treatment, this score might identify patients most likely to receive the greatest net benefit from aspirin (21). The Multi-Ethnic Study of Atherosclerosis (MESA) has recently shown in a pooled cohort that CACS might correctly identify asymptomatic patients for personalized allocation of aspirin in primary prevention of ASCVD (13). A patient with a CACS equal to or above 100 may be a good candidate for aspirin therapy for primary prevention, although the net expected benefit will likely be modest. In patients with a CACS of zero, the risk of bleeding is greater than the potential benefit, so aspirin therapy for primary prevention should be avoided.

In terms of MI prevention, low-dose aspirin has proven to be effective in inhibition of platelet aggregation and prevention of coronary thrombosis with potential reduction in mortality (22, 23). Magnitude of the CACS might have a role in reclassifying ASCVD risk, allowing identification of candidates likely to benefit from aspirin or other antiplatelet agents. Though evolving, the role of CACS in guiding antiplatelet therapy, especially to prevent MI, is still uncertain. Therefore, we conducted this study to find the causal relationship of CACS on MI and preventive effect of antiplatelet therapy in patients with suspected CAD.

## Materials and Methods

This prospective cohort study enrolled consecutive patients who underwent coronary CT scan at the Advanced Diagnostic Imaging Center (AIMC), Ramathibodi Hospital, Mahidol University, during November 2005 and November 2013. The study protocol conformed to the ethical guidelines of the 1975 Declaration of Helsinki and was approved by the Ethics Committee of the Faculty of Medicine, Ramathibodi Hospital, Mahidol University (# COA.MURA2019/758). Written informed consent was obtained from each participant before coronary CT study.

Inclusion criteria were: age > 18 years; being a patient who was asymptomatic, but had moderate to high ASCVD risk (24), or a symptomatic patient with suspected CAD. Exclusion criteria were severe asthma, high creatinine level (>1.5 mg/dL), severe seafood or contrast allergy, history of prior MI, coronary bypass or coronary stenting and previous gastrointestinal or intracranial bleeding.

### Baseline Clinical Evaluation

Data gathered from each patient included age, sex, ASCVD risk factors [e.g., smoking, diabetes mellitus (DM), hypertension and hypercholesterolemia], body mass-index (BMI, kg/m^2^), waist circumference (WC), and current medications including antiplatelet drugs (e.g., aspirin, and other P2Y12 inhibitors), anticoagulant and statin. Lab data collected included fasting plasma glucose (FPG), lipid profile, and serum creatinine. DM was defined as overnight FPG ≥ 126 mg/dL or taking anti-diabetic medication. Hypertension was defined as systolic BP ≥ 140 mmHg and/or diastolic BP ≥ 90 mmHg or taking anti-hypertensive medication. Smoking was classified as current smoking, previous smoking (stopped more than 1-month) or never smoked. Hypercholesterolemia was defined as total cholesterol ≥200 mg/dL or LDL-C ≥ 130 mg/dL, or taking a statin medication. Chronic kidney disease (CKD) was defined as an estimated GRF (eGFR) < 60 mL/min/1.73 m^2^. The eGFR (mL/min/1.73 m^2^) was calculated based on the CKD Epidemiology Collaboration (CKD-EPI)’s separate equations for men and women.

### Coronary Computed Tomography Angiography Protocol

The multidetector CT angiography (CTA) scans done during the study period used either a 64-slice scanner (Somatom Sensation 64 eco, Siemens, Forchheim, Germany) or a 320-slice scanner (Aquilion ONE, Toshiba, Tokyo, Japan) during 2005–2008 and 2008–2013, respectively. Coronary CT scan findings of interest included the CACS and degree of coronary stenosis by CTA. The CACS was calculated according to the Agatston method using a commercially available external work station (Vitrea fx 3.0.1, Vital Images, Minnesota, United States). Total CACS was calculated as the sum of the individual lesion scores in all coronary arteries and stratified into four groups: 0 = no identified plaque, 1–99 = mild atherosclerotic plaque, 100–399 = moderate atherosclerotic plaque, and ≥ 400 = extensive atherosclerotic plaque.

Degree of coronary stenosis was evaluated after injecting 70–90 mL of radiocontrast (Ultravist 370 mgI/mL, Bayer Healthcare Pharmaceuticals, Wayne, NJ, United States) via the right basilic vein through an 18-gauge intravenous catheter followed by a 20 mL saline flush at a flow rate of 5 mL/sec. Automated bolus tracking was used in order to synchronize the arrival of the contrast media and the scan. After a 4-s delay, images were obtained during an inspiratory breath hold of approximately 5–10 s.

Three-dimensional reconstruction and cross-sectional imaging measurements were performed; stenoses were assessed as none (0%), mild (≤ 25%), mild-moderate (25–50%), moderately severe (50–75%), or severe (≥ 75%). In this study, we reclassified the coronary lesions into three groups: normal coronary (0%), non-significant CAD (1- < 50%) and significant CAD (≥50%). All of these coronary CT findings were measured and interpreted by expert radiologists.

### Data Collection

All paper records (i.e., blood tests and full reports of coronary CT scans) were entered in duplicate into a digital file by two independent healthcare personnel (i.e., trained catheterization laboratory nurses or AIMC staff). Entered data were double-checked for consistency and readjusted for accuracy. Finally, all electronic databases were exported into an Excel spreadsheet for statistical analyses.

### Treatment and Long-Term Clinical Follow-Up

After the coronary CTA study, patients were treated as per their own physician’s discretion utilizing the CACS and coronary stenosis findings. Treatment options included antiplatelet and/or statin therapy combined with life-style and risk factor modification. Some patients also had cardiac stress test, invasive coronary angiography, and/or revascularization.

For this analysis, follow-up for treatment, clinical outcomes and vital status was captured through to December, 2019. Cross-sectional data was linked with several ongoing data sources including: (i) the electronic medical records Information Technology (IT) Department of Ramathibodi Hospital; (ii) 43-file data from the Strategy and Planning Division, Office of the Permanent Secretary, Ministry of Public Health (MoPH); (iii) the Information and Communication Technology (ICT) Center, MoPH, and (iv) Center Office for Healthcare Information, Health Systems Research Institute, MOPH. International Classification of Diseases, Tenth Revision (ICD-10) codes were clarified and reclassified into the outcomes of interest. These were fatal and non-fatal (i.e., non-ST-segment elevation or ST-segment elevation) MIs and bleeding events (i.e., gastrointestinal bleeding or intracranial bleeding), as listed by the standard ICD-10 codes.

### Statistical Analysis

Data was described by mean with standard deviation (mean ± *SD*) or frequency (*n*, %) for continuous or categorical data, respectively. Characteristics of patients who received or did not receive antiplatelet therapy were compared by Student’s *t*-test or Chi-square test for continuous or categorical data, respectively.

Mediation analysis was constructed based on the causal diagram (see Supplementary Figure 1), which considered the CACS as the independent variable, antiplatelet treatment as the mediator, and MI the outcome of interest. Generalized structural equation modeling was applied to construct models as follows:


$$Treatment_{i}=a_{0}+\sum_{i}CACS_{i}+\sum_{k}e_{k}z_{k}\;\left(patha\right)$$

MIi=b0+∑ic′iCACSi+∑ibiTreatmenti+∑kekzk(pathb)

*where CACS = 1–99 vs. 0, 100–399 vs. 0, ≥ 400 vs. 0; Treatment = yes vs. no; z_*k*_ = confounders.*

The mediation model was constructed by regression of the antiplatelet treatment on the CACS groups of 1–99, 100–399, and ≥ 400, given a CACS of 0 as the comparator (*path a*). Next, the MI outcome was regressed on the CACS groups and antiplatelet treatment (*path b*). The logit link function was then used for these two equations simultaneously, considering potential confounders whose *p*-values were less than 0.10 from univariate analysis in each model.

Mediation effect was then estimated using the product of coefficients (25). In addition, a 1,000-replication bootstrap was used to estimate average mediation effects (26), including both direct and indirect (mediation) effects along with their 95% confidence intervals (CI). All analyses were performed using STATA 17. A *p* < 0.05 was considered statistically significant.

## Results

Of 9,428 patients, 1,579 (16.7%) were excluded due to missing CACS data, development of MI/bleeding before performing CACS, or lost to follow-up leaving 7,849 eligible for analysis (see Supplementary Figure 2). The majority of patients were female (64.4%) with mean age and BMI of 59.3 ± 8.3 years old and 24.9 ± 3.6 kg/m^2^, respectively. Most patients had underlying ASCVD risk factors: hypertension (66.7%), hypercholesterolemia (53.8%), DM (27.1%), current or previous smoking (13.3%) and/or CKD (7.6%). Almost half (48.9%) of the patients had a CACS of zero, and only 6.3% had extensive atherosclerotic plaques with CACSs equal to or above 400. In terms of coronary stenosis, 39.8 and 39.1% of patients were normal or non-significant (<50% stenosis) CAD, respectively, and only 21.1% had significant (>50% stenosis) CAD.

Antiplatelet therapy was prescribed for about one-third (34.4%) of patients, and for most (88.4%) this was aspirin. Other medications used concurrently included anticoagulants (6.5%) and statins (72.5%). A subset of patients underwent invasive coronary angiography (10.1%) or revascularization (5.0%).

### Clinical Outcomes

During follow-up which averaged 9.9 ± 2.4 years, 176 participants developed a MI [2.24% (1.93, 2.59%)] and 545 [6.9% (6.39, 7.52)] developed a stroke, respectively. The composite endpoint of CV death, MI, or stroke was 8.9% (8.32%, 9.59). Bleeding events occurred in 221 [2.28% (2.46, 3.21)] subjects, mostly gastrointestinal [1.67% (1.39%, 1.97)] or intracranial [1.24% (1.0, 1.51)]. There was no fatal bleeding.

### Risk Factors and CT Angiography Findings Associated With Myocardial Infarction Occurrence

Patients who developed MI were older, more frequently male, had higher BMI and larger waist circumference, and more frequently had a ASCVD risk factor (i.e., ex/current smoking, hypertension, DM, low HDL-C, revascularization, statin use, and CKD) (Table 1). As noted, there was no association with hypercholesterolemia nor MI.

**TABLE 1:** Comparison characteristics between patients who diagnosis with MI and without MI.

Characteristics	Total	Diagnosis with MI		*p*-value
		Yes	No	
CACS, n (%)				
≥400	497 (6.3)	45 (9.1)	452 (90.9)	<0.001
100–399	996 (12.7)	58 (5.8)	938 (94.2)	
1–99	2,514 (32.0)	45 (1.8)	2,469 (98.2)	
0	3,842 (48.9)	28 (0.7)	3,814 (99.3)	
Antiplatelet, n (%)				
Yes	2,629 (33.5)	72 (2.7)	2,557 (97.3)	0.035
No	5,220 (66.5)	104 (2.0)	5,116 (98.0)	
Statin, n (%)				
Yes	5,690 (72.5)	165 (2.90)	5,525 (97.10)	<0.001
No	2,159 (27.5)	11 (0.51)	2,148 (99.49)	
Revascularization, n (%)				
Yes	393 (5.01)	80 (20.36)	313 (79.64)	<0.001
No	7,456 (94.99)	96 (1.29)	7,360 (98.71)	
Age, year, mean (SD)	59.3 (8.3)	63.4 (9.4)	59.2 (8.3)	<0.001
Sex, n (%)				
Male	2,798 (35.6)	93 (3.3)	2,705 (96.7)	<0.001
Female	5,051 (64.4)	83 (1.6)	4,968 (98.4)	
BMI, kg/m^2^, mean (SD)	24.9 (3.6)	25.7 (4.0)	24.9 (3.6)	0.005
Waist, inch, mean (SD)	34.4 (4.3)	35.5 (4.4)	34.4 (4.3)	0.001
Waist abnormal, n (%)				
Abnormal	3,991 (50.8)	88 (2.2)	3,903 (97.8)	0.820
Normal	3,858 (49.2)	88 (2.3)	3,770 (97.7)	
Smoking status, n (%)				
Ex/current smoke	1,034 (13.3)	39 (3.8)	995 (96.2)	<0.001
Never smoke	6,769 (86.7)	135 (2.0)	6,634 (98.0)	
Hypertension, n (%)				
Yes	5,236 (66.7)	166 (3.2)	5,070 (96.8)	<0.001
No	2,613 (33.3)	10 (0.4)	2,603 (99.6)	
DM, n (%)				
Yes	2,124 (27.1)	90 (4.2)	2,034 (95.8)	<0.001
No	5,725 (72.9)	86 (1.5)	5,639 (98.5)	
Hypercholesterolemia, n (%)				
Yes	4,213 (53.8)	93 (2.2)	4,120 (97.8)	0.862
No	3,619 (46.2)	82 (2.3)	3,537 (97.7)	
HDL-C, mean (SD)	49.0 (13.9)	44.4(13.5)	49.1 (13.9)	<0.001
eGFR, n (%)				
>60	7,247 (92.4)	143 (2.0)	7,104 (98.0)	<0.001
≤60	598 (7.6)	33 (5.5)	565 (94.5)	
CAD, n (%)				
≥50	1,658 (21.1)	104 (6.3)	1,554 (93.7)	<0.001
<50	3,066 (39.1)	50 (1.6)	3,016 (98.4)	
0	3,125 (39.8)	22 (0.7)	3,103 (99.3)	

*BMI, body mass index; CACS, coronary artery calcium score; CAD, coronary artery disease; DM, diabetes mellitus; eGFR, estimate glomerular filtration rate; HDL-C, high-density lipoprotein cholesterol.*

Extent of CACS was associated with the incidence of MI; CACSs of 0, 1–99, 100–399 and ≥ 400 had MI incidences of 0.7, 1.8, 5.8, and 9.1%, respectively (*p* < 0.001). Similarly, the severity of CAD was directly related to the incidence of MI; subjects with normal coronary artery, non-significant (<50%) CAD, and significant (≥50%) CAD had incidences of MI of 0.7, 1.6, and 6.3%, respectively (*p* < 0.001).

After adjustment for traditional risk factors (Table 2), CACSs of 100–399 and ≥ 400 were associated with MI [odds ratios (OR) (95% confidence interval; CI) of 3.14 (1.72, 5.72) and 3.22 (1.66, 6.25), respectively]. Other risk factors associated with MI included significant revascularization with OR of 11.64 (7.78, 17.43), hypertension with OR 3.46 (1.77, 6.77), and DM with OR 1.74 (1.25, 2.42). Former/current smoking trended toward, but did not reach statistical significance for MI development. Importantly, antiplatelet therapy was associated with a decreased occurrence of MI with OR 0.28 (0.19, 0.40), *p* < 0.001.

**TABLE 2:** Factors associated with MI diagnosis: multiple logistic regression.

Factors	OR	95% CI	*P*-value
CACS, n (%			
≥400	3.22	1.66, 6.25	<0.001
100–399	3.14	1.72, 5.72	<0.001
1–99	1.48	0.86, 2.56	0.156
0	1		
Antiplatelet			
Yes	0.28	0.19, 0.40	<0.001
No	1		
Statin			
Yes	0.75	0.06, 9.66	0.827
No	1		
HDL-C	0.97	0.91, 1.02	0.245
Revascularization			
Yes	11.64	7.78, 17.43	<0.001
No			
Smoking status			
Ex/current smoke	1.11	0.70, 1.68	0.604
Never smoke	1		
Hypertension			
Yes	3.46	1.77, 6.77	<0.001
No	1		
DM			
Yes	1.74	1.25, 2.42	0.001
No	1		
CAD, n %			
≥50	1.76	0.92, 3.40	0.089
<50	1.31	0.74, 2.33	0.352
0	1		

*BMI, body mass index; CACS, coronary artery calcium score; CAD, coronary artery disease; DM, diabetes mellitus; eGFR, estimate glomerular filtration rate; HDL-C, high-density lipoprotein cholesterol.*

### Risk Factors and CT Angiography Findings Associated With Antiplatelet Therapy

Patients who received antiplatelet therapy were older and more frequently male, overweight and had larger waist circumference. They were also characterized by ASCVD risk factors (including ex/current smoking, hypertension, DM, low HDL-C, hypercholesterolemia and CKD) (Supplementary Table 1).

Patients with higher CACSs tended to be treated with antiplatelets; i.e., 20.6, 39.3, 54.5, and 62.2% of patients received antiplatelet agents in the CACS 0, 1–99, 100–399 and ≥ 400 groups, respectively (*p* < 0.001). Similarly, antiplatelet therapy was more frequently prescribed to the patients with significant CAD; i.e., 19.8, 34.8, and 57.0% in groups with 0, < 50 and ≥ 50% stenosis, respectively (*p* < 0001).

### Mediation Analysis

A mediation analysis was applied by simultaneous construction of treatment and MI equations (Table 3 and Supplementary Table 2). For the treatment model, after adjusting for traditional risk factors, CACS 1–99, 100–399 and ≥ 400 groups were still directly related to frequency of antiplatelet therapy [the corresponding ORs (95% CI) were 1.35 (1. 15, 1.55), 1.49 (1.22, 1.81), and 1.57 (1.20, 2.05), respectively (*P* < 0.001)]. That is, patients with a positive (> 0) CACS were 35–57% more likely to receive antiplatelets than those whose CACS was 0. Other factors that were associated with antiplatelet therapy included degree of CAD stenosis, hypertension, DM, statin use, HDL-C, male gender and old age.

**TABLE 3:** Factors associated with receiving antiplatelet treatment and MI occurrence: a multivariate GSEM.

	Factor	OR	95% CI		*P*-value
Antiplatelet model	CACS 1-99	1.35	1.15	1.55	<0.001
	CACS 100-399	1.49	1.22	1.81	<0.001
	CACS ≥400	1.57	1.20	2.05	0.001
	Statin	3.55	1.75	7.20	<0.001
	HDL-C	0.98	0.96	0.99	0.015
	Age	1.03	1.02	1.04	<0.001
	Male	1.42	1.26	1.61	<0.001
	BMI	1.02	0.99	1.03	0.057
	Hypertension	1.99	1.74	2.29	<0.001
	DM	1.64	1.45	1.84	<0.001
	Hypercholesterolemia	1.11	0.99	1.24	0.064
	CAD<50	1.40	1.22	1.61	<0.001
	CAD≥50	2.31	1.92	2.78	<0.001
MI outcome model	CACS 1-99	1.48	0.86	2.55	0.156
	CACS 100-399	3.14	1.72	5.72	<0.001
	CACS≥400	3.22	1.66	6.25	0.001
	Antiplatelet	0.28	0.19	0.40	<0.001
	Statin	0.73	0.05	9.86	0.810
	HDL-C	0.96	0.91	1.02	0.245
	Revascularization	11.65	7.78	17.43	<0.001
	Ex or current smoke	1.11	0.74	1.67	0.604
	Hypertension	3.46	1.77	6.77	<0.001
	DM	1.73	1.25	2.42	0.001
	CAD<50	1.31	0.74	2.32	0.352
	CAD≥50	1.76	0.92	3.37	0.089

*BMI, body mass index; CACS, coronary artery calcium score; CAD, coronary artery disease; DM, diabetes mellitus; eGFR, estimate glomerular filtration rate; HDL-C, high-density lipoprotein cholesterol.*

For the MI model, results indicated that CACS was directly related to incidence of subsequent MI with ORs (95% CI) of 1.48 (0.86, 2.55), 3.14 (1.72, 5.72), and 3.22 (1.66, 6.25) for CACS 1–99, 100–399, and ≥ 400 groups (*p* < 0.001), respectively (Table 3). In addition, antiplatelet therapy significantly reduced the risk of MI development with OR of 0.28 (0.19, 0.40). Three other risk factors (revascularization, hypertension and DM) were also significantly associated with MIs.

**FIGURE 1:**
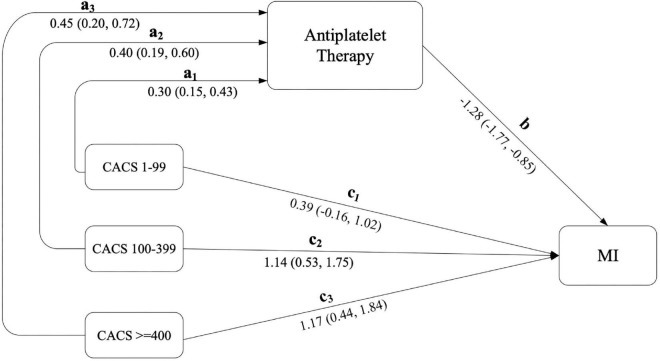
Estimation of direct and mediation effects of CACS on MI through antiplatelet therapy. CACS, independent factor; MI, interested outcome; antiplatelet therapy, mediator.

Mediation effects were further estimated using product of coefficients with a 1,000-replication bootstrap method (see Figure 1 and Table 4). This indicated that the effect of CACS level on MI occurrence was significantly reduced by antiplatelet treatment (the mediator) with ORs (95% CI) of 0.68 (0.52, 0.84), 0.60 (0.41, 0.78), and 0.56 (0.34, 0.77) for CACS of 1–99, 100–399, and ≥ 400, respectively.

**TABLE 4:** Estimation of direct and indirect effects of CAS on MI occurrence through antiplatelet treatments.

	Pathway	Coef.	95% CI		OR	95% CI	
Direct effect	CACS 1-99 → MI	0.39	−0.16	1.02	1.48	0.85	2.79
	CACS 100–399 → MI	1.14	0.53	1.75	3.14	1.70	5.75
	CACS≥400 → MI	1.17	0.44	1.84	3.23	1.55	6.31
Indirect effect	CACS 1–99 → Antiplatelet → MI	−0.38	−0.66	−0.18	0.68	0.52	0.84
	CACS 100–399 → Antiplatelet → MI	−0.51	−0.89	−0.25	0.60	0.41	0.78
	CACS≥400 → Antiplatelet → MI	−0.58	−1.08	−0.26	0.56	0.34	0.77

### Risk Factors Associated With Bleeding Events

Risk factors significantly associated with bleeding events were antiplatelet therapy, anticoagulant use, revascularization, old age, male gender, smoking status, hypertension, DM and CKD (Supplementary Table 3). After adjustment for co-variables, the only significant risk factors associated with bleeding were antiplatelet therapy (only aspirin) [OR 1.69 (1.25, 2.29)], anticoagulant [OR 1.89 (1.28, 2.79)], hypertension [OR 2.11 (1.40, 3.18)], male gender [OR 1.45 (1.10, 1.91)] and age [OR 1.05 (1.04, 1.07)] (Supplementary Table 4). Non-aspirin antiplatelet therapy and revascularization [1.51 (0.98, 2.33)] were not significantly associated with bleeding.

## Discussion

This cohort study of CTA patients was conducted with about 10 years of follow up. A mediation analysis demonstrated the significant direct effect of CACS on future MIs with the risk being about 1.5–3.2-folds higher in patients whose CACSs greater than zero. This risk was significantly reduced (about 32–44%) through the effects of antiplatelet therapy relative to patients who did not receive treatment. This indicated that antiplatelet therapy had a beneficial effect on the prevention of MIs in both asymptomatic patients with ASCVD risk factors or symptomatic patients with clinically suspected CAD. The study population in our cohort was heterogeneous and represented the majority of patients who are investigated for subclinical atherosclerosis in an attempt to prevent MI and CAD death. CACS, determined by coronary CT, may be a valuable noninvasive imaging modality to stratify risk of MI in asymptomatic patients and to guide implementation of antiplatelet therapy for primary prevention.

Our patient’s characteristics were quite similar to other cohorts who are studied with coronary CT, mainly middle-aged healthy individuals who are overweight and have multiple risk factors of ASCVD. After follow-up for almost 10 years, the incidence of MI in our cohort was 2.28%. That was similar to results in two previous cohort studies using CTA in patients with suspected CAD, whose incidences of MI were 2.3% at 5 year (27) and 3% at 10 year (28). Although these incidences seem low, they reflect real cohorts that include patients with appropriate treatment according to the CTA findings. Besides the statins that were prescribed for the majority of these patients, antiplatelet agents are another drug class proven to prevent MI and likely given to study subjects. One-third of our patients were prescribed at least one antiplatelet agent during the study period. Decision for prescribing antiplatelet therapy depended on an individual’s primary physician who would consider CACS and the degree of coronary stenosis, as well as any further investigations (such as cardiac stress test) and interventions (such as revascularization). As noted, there were some patients with a CACS greater than 100 or 400 who were not prescribed aspirin. About 10% of these patients had already taken anticoagulant and almost half of them were on statin treatment. The rest of patients were not prescribed as per the discretion of primary physicians and patient’s preference. However, it remains a controversy about the role of aspirin in term of routine primary prevention as recommended from the American and European guidelines because of increased bleeding risk (29, 30).

Our study confirmed that CACS was a strong independent predictor of future MI. The CACS cut-off of ≥ 100 was associated with increased risk of MI, and we suggest that it should be used to guide for personalized allocation of antiplatelet therapy in primary prevention. Our finding was similar to that in the report from the MESA group that participants with CACS ≥ 100 had more than a 4.2-fold higher risk for a CHD event and 2.8-fold higher risk for a CVD event compared with those with CACS = 0 (31). However, the studies differed in that the outcomes of the MESA study also included nonfatal and fatal strokes. Our study focused only on MI (and antiplatelet therapy) because it is the most devastating CAD event and may cause sudden death. In terms of stroke, the benefits of aspirin as a primary prevention are still uncertain (32–34). A recent large meta-analysis found that there was no benefit of aspirin for stroke primary prevention, and that it was associated with an increased risk of major gastrointestinal and hemorrhagic stroke (34). In that study, aspirin was associated with a modest reduction in non-fatal MI. We believe that this controversy makes it even more important to use the non-invasive quantitation of CACS to improve traditional risk scores and guidance regarding treatment.

Even though we found an association between CACS and occurrence of future MI, the impact of coronary calcium on the natural history of CAD remains uncertain. There are potentially divergent prognostic implications of CACSs. Some researchers suggest that large calcification is a marker of coronary plaque stabilization and associated with stable angina (and reduced risk of ACS events) (35, 36). Moreover, the density and pattern of calcification within a plaque may impact the risk of future MI (37, 38). In general, more coronary calcium reflects a prior coronary plaque rupture followed by vessel-wall healing and calcification (39). CACS positivity also may be a surrogate measure of non-calcified atherosclerosis in coronary arteries and increased risk of MI (4). Therefore, CACS may be a good surrogate marker of future MI and thus a potential screening tool for guiding antiplatelet therapy as a primary prevention. In this vein, we observed that patients with non-calcified plaques or zero CACS also can develop MI in the subsequent decade, but the risk of this was much less than that associated with highly calcified plaques. The modest benefit of MI prevention by prescribing antiplatelet therapy in patients with zero CACS must be considered against the risk of bleeding, and should be carefully considered in the individual patient. Following CACS over time is another option for surveillance of these patients. There is evidence that progression of CAC is associated with CVD (4, 40).

From mediation analysis, we found that the likelihood of prescribing antiplatelet therapy was partly driven by the CACS. There is evidence that CACS is equivalent to coronary stenosis measured by coronary CTA in predicting mortality and MI in asymptomatic adults (41). After treating with antiplatelet therapy, as expected, the risk of a future MI was reduced by 18–24%, in proportion to the magnitude of the CACS. Benefit was seen even in the group with CACS 1–99, and became clearer with CACSs equal to or above 100. Although antiplatelet therapy seems to be effective to prevent MI for the whole range of plaque pathology, its use must be balanced against the risk and consequences of bleeding. In our study, the incidence of bleeding was 2.28%. This was comparable to that found in a large meta-analysis in terms of using aspirin as a primary prevention (34). The majority of bleeding events in our study population were non-fatal and mostly in the GI tract. Several previous studies suggested that CACS ≥ 100 had favorable risk/benefit balance for aspirin use while participants with zero CACS were assessed to receive net harm from the therapy (13, 21, 31). All of these studies used only aspirin for primary prevention of CVD. It is known that aspirin causes mucosal ulcers and is associated with upper GI side effects, including gastritis, ulcers and bleeding, due to its inhibition on prostaglandin synthesis (42, 43). In patients at high risk for ASCVD with high CACS, combined administration of a proton-pump-inhibitor with aspirin or a switch to an alternative P2Y12 inhibitor, especially clopidogrel, seems to be reasonable and effective therapy for prevention without excessive gastrointestinal bleeding side effects (44). For intracranial bleeding, it is difficult to predict who will develop this. In our study, old age, male gender, anticoagulant use and hypertension were the risk factors for bleeding. Therefore, antiplatelet drugs should only be given to these patients with caution while making certain that their blood pressure remains in good control.

Patients who have a CACS of 0 or < 100, or non-significant coronary stenosis, still have a chance of having a silent non-calcified plaque or progression of an atherosclerotic plaque which later may result in symptomatic CAD or MI (45). A recent study report stated that the frequency of non-calcified atherosclerotic plaques in the coronary arteries of patients with CACSs of zero was 9.3% (46). Such individuals who develop CVD events have been shown to have a higher prevalence of potentially modifiable ASCVD risk factors, such as DM and smoking. In our study, the incidence of MI was about 2.4% in these groups. Even though antiplatelet therapy is not recommended in those low-risk groups because the chance of bleeding may outweigh benefit, they should be prescribed statin therapy (if LDL > 70 mg/dL) and recommended to change life-style and modify risk factors, as these strategies may be effective and adequate for primary prevention of MI without any bleeding risk (20, 47).

In summary, our mediation analysis showed that CACS was directly related to occurrence of MI and helped to guide use of antiplatelet therapy for primary prevention. It is necessary to mention that this mediation analysis demonstrated association, but not causation, and did not confirm the efficacy of antiplatelet therapy to prevent MI according to the magnitude of CACS. These unanswered questions will need further a randomized controlled study to resolve.

### Limitations

Although we studied the largest Asian population cohort to have these CAD studies and long-term follow-up, we acknowledge several limitations of our study. First, there were some patients (less than 10%) who were lost to follow-up. These patients usually were not covered by any health care insurance (mostly self-pay) and so were not picked up in our follow-up databases. There was no recorded data of patient’s symptoms, in which risk of MI may not be the same in symptomatic and asymptomatic individuals. However, most symptoms in our participants (i.e., chest pain on exertion or dyspnea on exertion) were mild and not alike MI. The clinical outcomes of interest (MI and bleeding events) were identified by ICD-10 codes and were not completely adjudicated by our investigators, leading to the possibility of some misclassification. Receipt of antiplatelet treatment by patients was confirmed by the bill of reimbursement, although patient compliance was not directly assessed. Furthermore, there was a possibility that concomitant drugs may have been used which increased the chance of bleeding, such as anticoagulants or non-steroidal inflammatory drugs (NSAIDs). Finally, most of our subjects were of Asian ethnicity, suggesting care must be taken when applying these results to other populations.

## Conclusion

This was the first mediation analysis to demonstrate a causal relationship between CACS and the risk of future MI, and that the beneficial effect of antiplatelet therapy to prevent MI depends on the magnitude of CACS. Patients who had higher CACSs were likely to be treated with antiplatelet therapy. Antiplatelet therapy as guided by the CACS may prevent future MIs and minimize bleeding risk. The preventive effect of antiplatelet therapy was clearly demonstrated in subjects with CACSs equal to or above 100. However, this benefit was partially offset by an increased incidence of bleeding in high-risk patients, especially with aspirin use. These results provide a rationale for a future randomized controlled study to validate the efficacy of antiplatelet therapy for primary prevention in other populations with varying degrees of CACS.

## Data Availability Statement

The raw data supporting the conclusions of this article will be made available by the authors, without undue reservation.

## Ethics Statement

The studies involving human participants were reviewed and approved by the Ethics Committee of the Faculty of Medicine, Ramathibodi Hospital, Mahidol University (# COA.MURA2019/758). The patients/participants provided their written informed consent to participate in this study.

## Author Contributions

TL initiated research question and wrote research proposal and the entire manuscript. SJ analyzed coronary computed tomography angiography (CCTA). NU collected data base and performed statistical analysis. SR managed data and performed statistical analysis. AT supervised statistical analyses and revised manuscript. JL provided funding and initiated for the CCTA project. All authors contributed to the article and approved the submitted version.

## Conflict of Interest

The authors declare that the research was conducted in the absence of any commercial or financial relationships that could be construed as a potential conflict of interest.

## Publisher’s Note

All claims expressed in this article are solely those of the authors and do not necessarily represent those of their affiliated organizations, or those of the publisher, the editors and the reviewers. Any product that may be evaluated in this article, or claim that may be made by its manufacturer, is not guaranteed or endorsed by the publisher.
