# Supplementary material for: Causal Relationship of Coronary Artery Calcium on Myocardial Infarction and Preventive Effect of Antiplatelet Therapy
Source: Front Cardiovasc Med. 2022 Apr 27;9:871267. doi: 10.3389/fcvm.2022.871267 (PMC9091507; doi:10.3389/fcvm.2022.871267)
Supplement: Supplementary file 1 [file Data_Sheet_1.pdf]

Supplemental Table

Supplemental Table 1. Comparison of characteristics between patients who did and did not receive antiplatelet

Supplemental Table 2. Factors associated with receiving antiplatelet: Multiple logistic regression

Supplement Table 3. Comparison of characteristics between patients who have bleeding and did not have bleeding

Supplemental Table 4. Factors associated with bleeding: Multiple logistic regression

Supplemental Table 1. Comparison of characteristics between patients who did and did not receive antiplatelet

Characteristic	Antiplatelet Therapy		p-value
	Given	Not given	
CACS, n (%)			
≥ 400	309 (62.2)	188 (37.8)	<0.001
100 - 399	543 (54.5)	453 (45.5)	
1 - 99	987 (39.3)	1,527 (60.7)	
0	790 (20.6)	3,052 (79.4)	
Degree of CAD stenosis, n (%)			
≥50	945(57.0)	713 (43.0)	<0.001
<50	1,066(34.8)	2,000 (65.2)	
0	618(19.8)	2,507 (80.2)	
Statin, n %			
Yes	2,521 (42.9)	3,350 (57.1)	<0.001
No	153 (5.4)	2,681 (94.6)	
Age, years, mean (SD)	62.1 (7.9)	57.9 (8.1)	<0.001
Sex, n (%)			
Male	1,134 (40.5)	1,664 (59.5)	<0.001
Female	1,495 (29.6)	3,556 (70.4)	
BMI, kg/m^2, mean (SD)	25.5 (3.6)	24.7 (3.6)	<0.001
Waist, inches, mean (SD)	35.2 (4.3)	34.0 (4.2)	<0.001
Waist abnormal, n (%)			
Abnormal	1,413 (35.4)	2,578 (64.6)	<0.001
Normal	1,216 (31.5)	2,642 (68.5)	
Smoking status, n (%)			
Ex/current smoke	429 (41.5)	605 (58.5)	<0.001
Never smoke	2,184 (32.3)	4,585 (67.7)	
Hypertension, n (%)			
Yes	2,250 (43.0)	2,986 (57.0)	<0.001
No	379 (14.5)	2,234 (85.5)	
DM, n (%)			
Yes	1,069 (50.3)	1,055 (49.7)	<0.001
No	1,560 (27.2)	4,165 (72.8)	

Supplemental Table 1 (cont'd). Comparison of characteristics between patients who did and did not receive antiplatelet

Characteristic	Antiplatelet Therapy		p-value
	Given	Not given	
HDL-C, mean (SD)	46.6 (13.6)	50.2 (13.9)	<0.001
Hypercholesterolemia, n (%)			
Yes	1,650 (39.2)	2,563 (60.8)	<0.001
No	973 (26.9)	2,646 (73.1)	
eGFR, n (%)			
> 60	2,316 (32.0)	4,931 (68.0)	<0.001
≤ 60	313 (52.3)	285 (47.7)	

BMI: body mass index, CACS: coronary artery calcium score, CAD: coronary artery disease, DM: diabetes mellitus, eGFR: estimated glomerular filtration rate, HDL-C: high-density lipoprotein cholesterol

Supplemental Table 2. Factors associated with receiving antiplatelet: Multiple logistic regression

Factors	OR	95% CI	P-value
CACS			
≥ 400	1.57	1.21, 2.02	0.001
100 – 399	1.48	1.22, 1.79	<0.001
1 – 99	1.35	1.17, 1.55	<0.001
0	1		
Statin			
Yes	5.74	2.93, 11.93	<0.001
No	1		
HDL-C	0.98	0.95, 1.00	0.082
Age	1.03	1.02, 1.04	<0.001
Sex			
Male	1.39	1.24, 1.57	<0.001
Female	1		
BMI, kg/m^2	1.01	0.99, 1.03	0.121
Hypertension			
Yes	2.11	1.84, 2.42	<0.001
No	1		
DM			
Yes	1.65	1.47, 1.87	<0.001
No	1		
Hypercholesterolemia			
Yes	1.08	0.96, 1.21	0.162
No	1		
CAD			
≥50	2.24	1.87, 2.68	<0.001
<50	1.38	1.20, 1.58	<0.001
0	1		

Abbreviations as in supplemental Table 1.

Supplemental Table 3. Comparison of characteristics between patients who have bleeding and did not have bleeding

Characteristics	Total	Bleeding		p-value
		Yes	No	
Antiplatelet, n %				
ASA	2,398 (30.6)	118 (4.9)	2,280 (95.1)	<0.001
Other	315 (4.0)	12 (3.8)	303 (96.2)	
None	5,136 (65.4)	91 (1.8)	5,045 (98.2)	
Anticoagulant, n %				
Yes	512 (5.88)	38 (7.48)	470 (92.52)	<0.001
No	8,193 (94.12)	183 (2.49)	7,158 (97.51)	
Revascularization, n %				
Yes	393 (5.01)	31 (7.89)	362 (92.11)	<0.001
No	7,456 (94.99)	190 (2.55)	7,266 (97.45)	
Age, year, mean SD	59.3 (8.3)	63.9 (9.3)	59.2 (8.2)	<0.001
Sex, n %				
Male	2,798 (35.6)	103 (3.7)	2,695 (96.3)	0.001
Female	5,051 (64.4)	118 (2.3)	4,933 (97.7)	
BMI, kg/m^2, mean SD	24.9 (3.6)	24.8 (3.5)	24.9 (3.6)	0.686
Waist, inch, mean SD	34.4 (4.3)	34.9 (4.2)	34.4 (4.3)	0.057
Waist abnormal, n %				
Abnormal	3,991 (50.8)	113 (2.8)	3,878 (97.2)	0.932
Normal	3,858 (49.2)	108 (2.8)	3,750 (97.2)	
Smoking status, n %				
Ex or current smoke	1,034 (13.3)	43 (4.2)	991 (95.8)	0.005
Never smoke	6,769 (86.7)	176 (2.6)	6,593 (97.4)	
Hypertension, n %				
Yes	5,236 (66.7)	192 (3.7)	5,044 (96.3)	<0.001
No	2,613 33.3	29 1.1	2,584 98.9	
DM, n %				
Yes	2,124 (27.1)	91 (4.3)	2,033 (95.7)	<0.001
No	5,725 (72.9)	130 (2.3)	5,595 (97.7)	

Supplemental Table 3 (cont'd). Comparison of characteristics between patients who have bleeding and did not have bleeding

Characteristics	Total	Bleeding		p-value
		Yes	No	
Hypercholesterolemia, n %				
Yes	4,213 (53.8)	109 (2.6)	4,104 (97.4)	0.226
No	3,619 (46.2)	110 (3.0)	3,509 (97.0)	
eGFR, n %				
> 60	7,247 (92.4)	183 (2.5)	7,064 (97.5)	
≤ 60	598 (7.6)	38 (6.4)	560 (93.6)	<0.001

Abbreviations as in supplemental Table 1.

Supplemental Table 4. Factors associated with bleeding: Multiple logistic regression

Factors	OR	95% CI	P-value
Antiplatelet drug			
ASA	1.69	1.25, 2.29	0.001
Other	1.16	0.62, 2.19	0.635
None	1		
Anticoagulant			
Yes	1.89	1.28, 2.79	0.001
No	1		
Revascularization			
Yes	1.51	0.98, 2.33	0.065
No	1		
Age, year	1.05	1.04, 1.07	<0.001
Sex			
Male	1.38	1.04, 1.81	0.024
Female	1		
Hypertension			
Yes	2.01	1.33, 3.04	0.001
No	1		
