# Supplementary figures and images for: Causal Relationship of Coronary Artery Calcium on Myocardial Infarction and Preventive Effect of Antiplatelet Therapy
Source: Front Cardiovasc Med. 2022 Apr 27;9:871267. doi: 10.3389/fcvm.2022.871267 (PMC9091507; doi:10.3389/fcvm.2022.871267)

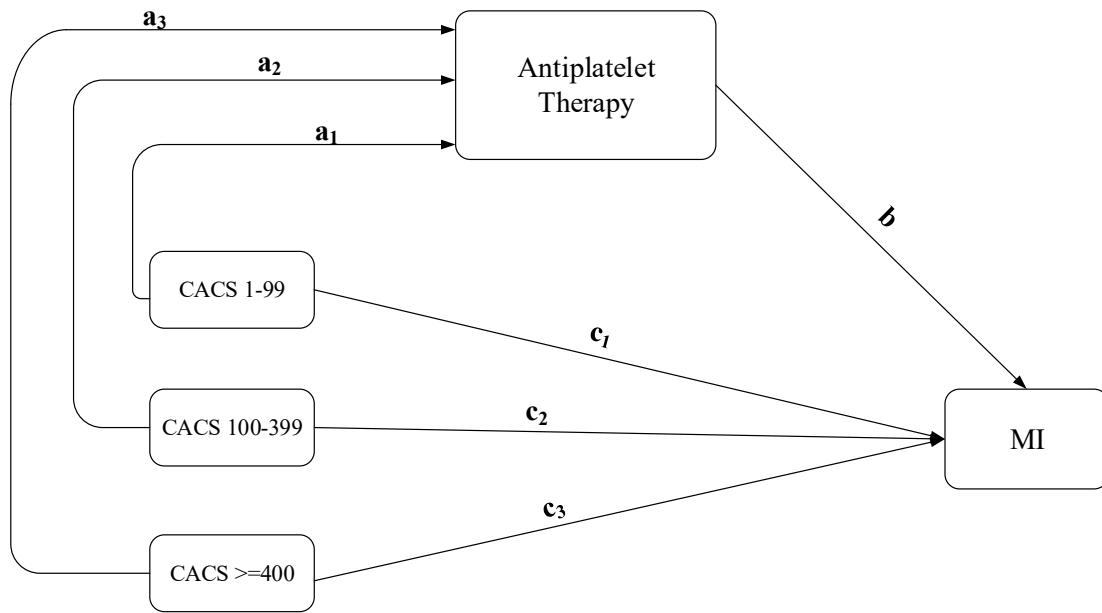

**Supplemental Figure 1.** A causal diagram of CACS, antiplatelet therapy and MI.

Supplement: Supplementary file 2 [file Image_1.pdf]

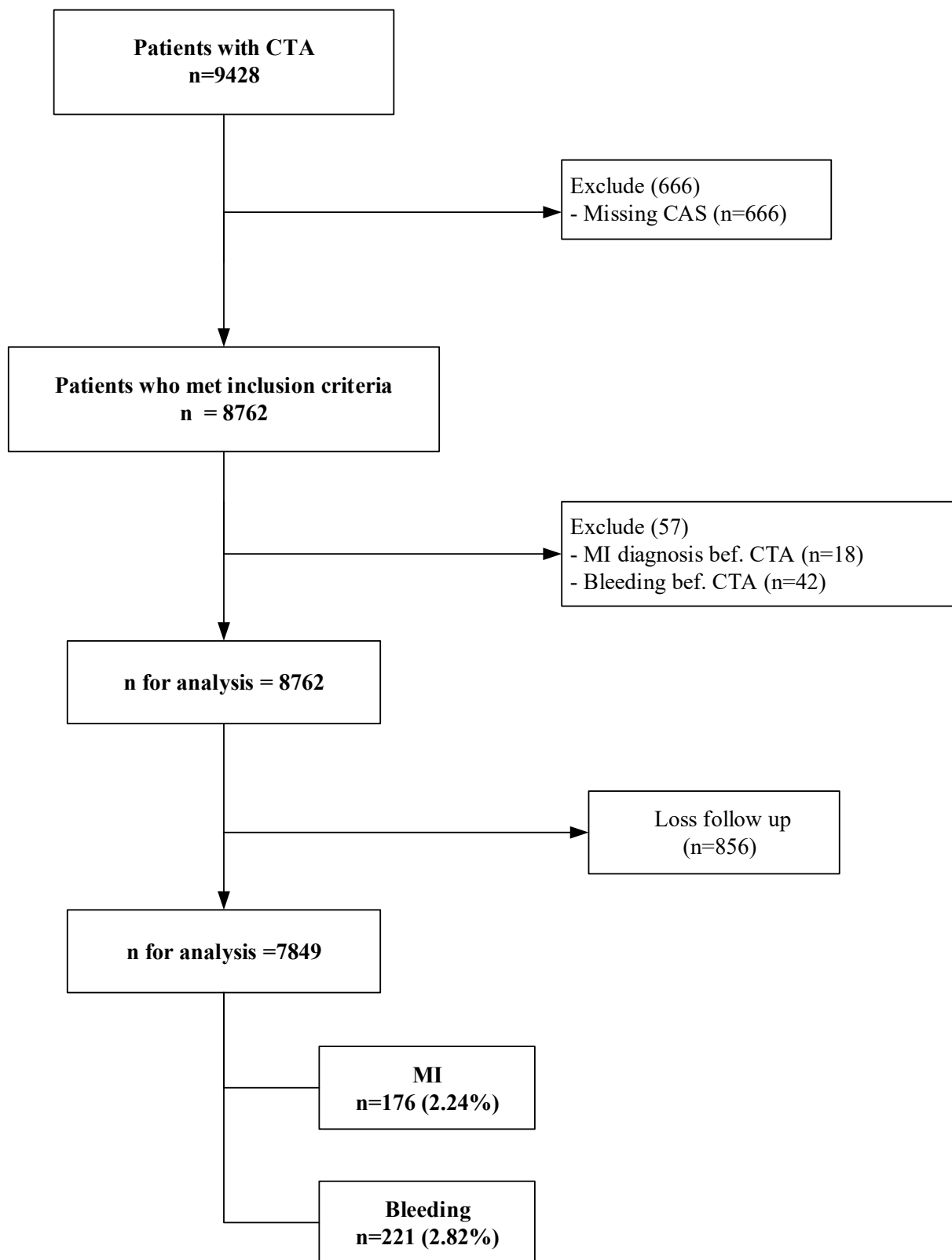

**Supplemental Figure 2.** Summary numbers of included subjects

Supplement: Supplementary file 3 [file Image_2.pdf]
